# Using the Nutrition Facts Label to Make Food Choices Is Associated with Healthier Eating among 8th and 11th-Grade Students: An Analysis of Statewide Representative Data from the 2019–2020 Texas School Physical Activity and Nutrition Survey

**DOI:** 10.3390/nu16020311

**Published:** 2024-01-20

**Authors:** Christopher D. Pfledderer, Nalini Ranjit, Adriana Pérez, Raja I. Malkani, Augusto César Ferreira De Moraes, Ethan T. Hunt, Carolyn L. Smith, Deanna M. Hoelscher

**Affiliations:** 1 Department of Health Promotion and Behavioral Sciences, The University of Texas Health Science Center at Houston (UTHealth) School of Public Health in Austin, Austin, TX 78701, USA; nalini.ranjit@uth.tmc.edu (N.R.); ethan.t.hunt@uth.tmc.edu (E.T.H.); deanna.m.hoelscher@uth.tmc.edu (D.M.H.); 2 Michael & Susan Dell Center for Healthy Living, The University of Texas Health Science Center at Houston (UTHealth) School of Public Health in Austin, Austin, TX 78701, USA; adriana.perez@uth.tmc.edu (A.P.); raja.i.malkani@uth.tmc.edu (R.I.M.); carolyn.l.smith@uth.tmc.edu (C.L.S.); 3 Department of Biostatistics and Data Science, The University of Texas Health Science Center at Houston (UTHealth) School of Public Health in Austin, Austin, TX 78701, USA; 4 Department of Epidemiology, The University of Texas Health Science Center at Houston (UTHealth) School of Public Health in Austin, Austin, TX 78701, USA; augusto.cesar.ferreirademoraes@uth.tmc.edu

**Keywords:** nutrition, nutrition facts, food labels, healthy usingeating, adolescents, Texas SPAN

## Abstract

Background: Nutrition labels are a tool to inform and encourage the public to make healthier food choices, but little information is available about use in multi-ethnic adolescent populations in the U.S. The purpose of this study was to examine associations between the level of nutrition label usage and healthy/unhealthy eating behaviors among a statewide representative sample of 8th and 11th-grade students in Texas. Methods: We analyzed cross-sectional associations between the Nutrition Facts label use and eating behaviors from a statewide sample of 8th and 11th-grade students in Texas, (*n* = 4730, weighted *n* = 710,731, mean age = 14.7 ± 1.6 years; 49% female, 51% Hispanic), who completed the 2019–2020 Texas School Physical Activity and Nutrition (TX SPAN) survey. Students self-reported their level of nutrition label usage to make food choices (5-point Likert scale from “Never” to “Always”) and previous day consumption of 26 food items (13 healthy, 13 unhealthy). The 26 food items were used to calculate a Healthy Eating Index (HEI) score (0–100), a Healthy Foods Index (HFI) score (0–100), and an Unhealthy Foods Index (UFI) score (0–100). Weighted linear regression models were employed to examine the associations between self-reported use of nutrition labels to make food choices and HEI, HFI, and UFI scores. Marginal predicted means of HEI, HFI, and UFI scores were calculated post hoc from linear regression models. The odds of consuming specific individual food items for nutrition label usage were also calculated from weighted logistic regression models. All linear and logistic regression models were adjusted for grade, sex, Body Mass Index (BMI), race/ethnicity, economic disadvantage, and percentage of English language learners by school. Results: A total of 11.0% of students reported always/almost always using nutrition labels to make food choices, 27.9% reported sometimes using them, while 61.0% indicated they never/almost never used nutrition labels to make food choices. The average HEI score among students in the sample was 47.7 ± 5.9. Nutrition Facts label usage was significantly and positively associated with HEI (b = 5.79, 95%CI: 4.45, 7.12) and HFI (b = 7.28, 95%CI:4.48, 10.07), and significantly and negatively associated with UFI (b = −4.30, 95%CI: −6.25, −2.34). A dose–response relationship was observed between nutrition label usage and HEI, HFI, and UFI scores, such that the strength of these associations increased with each one-point increase in nutrition label usage. Students who reported using nutrition labels always/almost always to make food choices had significantly higher odds of consuming healthy foods including baked meat, nuts, brown bread, vegetables, whole fruit, and yogurt (OR_range_ = 1.31–3.07), and significantly lower odds of consuming unhealthy foods including chips, cake, candy, and soda (OR_range_ = 0.48–0.68) compared to students who reported never/almost never using the Nutrition Facts label. Conclusions: Using the Nutrition Facts labels to make food choices is beneficially associated with healthy and unhealthy eating among 8th and 11th-grade students, although the proportion of students using nutrition labels to make their food choices was low. Public health efforts should be made to improve nutrition literacy and encourage nutrition label use among secondary students in the United States.

## 1. Background

The Nutrition Labeling and Education Act of 1990 made accessible standardized nutrition information on most packaged foods available to consumers in the United States. Today, most packaged foods regulated by the United States Food and Drug Administration (US FDA) have nutrition labels (Nutrition Facts labels), which provide information about serving size, calories, and nutrients including calcium, fiber, total fat, cholesterol, and sodium. This information can be used to help consumers compare food items and potentially make healthier food choices. Most recently, the US FDA updated the Nutrition Facts label, which now displays serving sizes and calories in a larger and bolder font, requires added sugars to be included in grams and a percent Daily Value, updated the list of nutrients required/permitted on the label, and updated footnotes to better explain percent Daily Value information [1,2].

Most research on nutrition label usage in the US has been conducted with adult populations. Research shows that nearly 80% of US adults use nutrition labels to inform buying decisions, and the usage of labels has increased each year since 2005–2006 [3]. Moreover, nutrition label use has been shown to be associated with healthier nutrient consumption [4], including the consumption of less energy, saturated fat, carbohydrates, sugar [5], and sodium [6], overall dietary intake [7], and lower long-term diabetes risk [8]. While the research conducted with adult populations is promising, less is known about nutrition label usage among younger populations, including adolescents.

Adolescence marks an important period in the transition from childhood to adulthood, accompanied by changes in lifestyle and health behaviors, including increased autonomy in food choices [9,10,11]. Additionally, nearly a quarter of adolescents in the US have obesity [12]. In this context, the use of nutrition labels to make informed dietary choices becomes of particular interest, as it presents an opportunity to understand the extent to which adolescents engage with nutritional information and how it influences their dietary patterns. The few studies that have been conducted on nutrition label use among adolescents in the US have shown conflicting results. For example, one study found that almost 80% of the adolescent sample reported reading nutrition labels [13], which is similar to what is currently reported for adult populations, although results showed that nutrition label reading was not associated with a healthier diet. Conversely, another study using data from the 2005–2006 National Health and Nutrition Examination Survey (NHANES) found that less than 25% of adolescents used nutrition labels to make food choices [14]. Given the paucity of research on adolescent nutrition label usage and subsequent associations with dietary behavior, there is a critical need for more studies on this topic. Furthering our understanding of the link between nutrition label use and dietary behavior among adolescents may shed light on potentially effective obesity-related prevention programs that may leverage systems-based policy in the US for this age group, as has been suggested in other parts of the world [15,16,17].

The purpose of this study is to examine associations between the level of nutrition label usage and healthy/unhealthy eating behaviors among a statewide representative sample of 8th and 11th-grade students in Texas who took part in the 2019–2020 Texas School Physical Activity and Nutrition (Texas SPAN) survey. While a similar study on nutrition label usage and healthy eating was conducted using the 2008–2009 Texas SPAN data [18], the current study uses data collected a year after the US FDA released a new “Nutrition Facts” label for food items in 2018 [1,2]. This gives us the opportunity to extend the findings of the previous study and highlight the prevalence of adolescents using the new labels as well as how this usage is associated with eating habits. In this study, we also explore how nutrition label usage influences the consumption of 26 individual food items, which may provide more detailed information on how nutrition labels could be used to promote healthy eating and dissuade unhealthy eating. By addressing these questions, this research contributes to our understanding of adolescent nutrition and informs strategies for promoting healthier dietary behaviors. Additionally, it highlights the importance of education and policy interventions aimed at empowering adolescents to make informed food choices that support their long-term health and well-being.

## 2. Methods

### 2.1. Study Design

This cross-sectional study uses data from the 2019–2020 Texas Survey of Physical Activity and Nutrition (SPAN) surveillance study, a statewide survey of behaviors related to diet and physical activity, and accompanied by objective anthropometric measurements. SPAN survey and anthropometric data are obtained from a representative sample of 2nd, 4th, 8th, and 11th-grade students in the state of Texas, using the Texas Education Agency enrollment data as the sampling frame, and a complex sampling design. This study uses data collected from 8th and 11th graders during the 2019–2020 academic year. The Committee for the Protection of Human Subjects at the University of Texas Health Science Center at Houston (UTHealth Houston) (HSC-SPH-18-0432), the Texas Department of State Health Services Institutional Review Board, and local school district review committees reviewed and approved all study-related activities. Detailed descriptions of the overall Texas SPAN study have been reported elsewhere [19,20,21], but specific information for participants and measures used in this study are provided below.

### 2.2. Participants, Data Collection, and Sampling

The Texas SPAN survey is a self-administered survey questionnaire. Survey items include questions about demographic characteristics, nutrition, physical activity, screen time, and dental habits. In addition to questionnaire items, Texas SPAN collects objective measures of height and weight used to calculate Body Mass Index (BMI). The stratified, multi-stage sampling of the Texas SPAN survey included representative data for the state, eight public health regions, and border/non-border areas by using data obtained from the Texas Education Agency (TEA) on public school enrollment to create the sampling frame (weighting structure) for the study. The total number of 8–11th-grade students included in the 2019–2020 SPAN survey was 4730, representing a weighted sample of 710,731 8th and 11th-grade students across Texas.

### 2.3. Measures

The following section provides details about the specific measures from the Texas SPAN survey used for this study. A full copy of the Texas SPAN survey has been published online for further information [21].

#### 2.3.1. Measures of Dietary Behavior

Dietary behaviors were assessed with a series of questions that assessed the frequency of prior day consumption of each of multiple food items. Surveys were administered Tuesdays to Fridays, to ensure that only weekday consumption was assessed. These questions were preceded by the statement, “Think about everything you ate or drank (at home, school, a friend’s house, or anywhere else) from the time you got up yesterday morning until the time you went to sleep last night”. Students were then provided with a list of 26 typical food items (13 healthy, 13 unhealthy) and were asked to record the number of times they ate/drank those food items, with response categories of 0, 1, 2, or 3 or more times. The 13 healthy items were baked meat, nuts, brown rice, brown bread, starchy, orange, green, and other vegetables, beans, whole fruit, fruit juice, plain milk, and yogurt. The 13 unhealthy items were red meat, fried meat, white rice, white bread, chips, frozen desserts, cake, candy, flavored milk, punch (fruit-flavored drinks), soda, sweetened caffeinated beverages, and energy drinks. All dietary questions used in the survey have been shown to be both valid and reliable [22]. These items were used to calculate the SPAN Healthy Eating Index (SPAN HEI), which served as the primary outcome for this study, as follows: responses to the 13 healthy food items were summed and responses to the 13 unhealthy food items were reversed coded and summed. These two sums were combined and rescaled from 0–100, with a higher score indicating a healthier diet. Two additional indices were created to assess dietary behaviors. A Healthy Foods Index (HFI) score was created by summing responses to the 13 healthy food items and scaling the sum to a 0–100 range, where a higher score indicates a healthier diet. The Unhealthy Foods Index (UFI) was similarly created from responses to the 13 unhealthy food items. A higher score on the UFI indicates an unhealthier diet, as individual unhealthy food items were not reverse coded in the calculation of the UFI. Finally, for analyses evaluating consumption of individual food items, responses were dichotomized into 0 (reported not eating the food item on the day prior) and 1 (reported eating the food item at least once on the day prior).

#### 2.3.2. Nutrition Facts Label Usage

Nutrition Facts label usage served as the primary predictor for this study and was assessed with a single question that asked, “Do you use food labels (Nutrition Facts) to make food choices”? Response options were presented as a 5-point Likert scale, including “Never”, “Almost never”, “Sometimes”, “Almost always”, and “Always”. A picture of the Nutrition Facts label was provided alongside the question as a visual aid. For the logistic regression analyses, response options were collapsed into three categories, which were Never/Almost never, Sometimes, and Almost always/Always.

#### 2.3.3. Weight Status

Objective measures of height and weight were used to calculate Body Mass Index (BMI) for each student using the SAS code provided by the Centers for Disease Control and Prevention (CDC) [23] and were further classified as obesity, overweight, and healthy weight, using the CDC growth charts and current recommendations [24]. Height was recorded in centimeters with a stadiometer and weight was recorded in kilograms using a calibrated top-loading scale. Detailed methods have been reported elsewhere [20].

#### 2.3.4. Socio-Demographic Variables

The sex of students was determined with a single question that asked, “What are you?” and included the response options “Male” and “Female”. Race/ethnicity was determined with a single question that asked, “How do you describe yourself?”. Responses included “Black or African American”, “Latino, Hispanic, or Mexican American”, “White, Caucasian, or Anglo”, “Asian (from India or Pakistan)”, “Asian (not from India or Pakistan)”, “American Indian or Alaska Native”, Native Hawaiian or Other Pacific Islander”, and “Other (write in)”. Economic disadvantage, assessed at the school and grade levels as the percentage of students that are economically disadvantaged, was based on data provided by the TEA [25]. Economic disadvantage is defined by the TEA to indicate children qualifying for free or reduced meals under the National School Lunch and Child Nutrition Program and/or families with an annual income at or below the United States poverty threshold. A second sociodemographic measure, also assessed at the school and grade levels, was the percentage of students who were reported as having limited English proficiency (LEP) based on data provided by the TEA.

### 2.4. Statistical Analysis

The unweighted counts and weighted percentages of all descriptive characteristics were computed for the total sample and 8th and 11th graders separately. Weighted linear regression models were employed to examine the associations between self-reported use of nutrition labels to make food choices and HEI, HFI, and UFI scores. Marginal predicted means of HEI, HFI, and UFI scores were calculated post hoc from linear regression models. The odds of consuming specific individual food items based on Nutrition Facts label usage were also calculated from weighted logistic regression models and significance values were corrected with Bonferroni’s method. All linear and logistic regression models were adjusted for grade, sex, race/ethnicity, percent that were economically disadvantaged, percent limited English proficiency, and Body Mass Index (BMI). STATA’s ‘svyset’ prefix command was used to account for the complex sampling plan of the Texas SPAN survey data and weighted analyses used the Taylor Series Linearization variance estimation. All analyses were carried out with STATA v18.0 (StataCorp LP, College Station, TX, USA) and a type I error level of 0.05 was used for linear regression models. To account for multiple testing with the 26 weighted logistic regression models, a type I error level of 0.002 was used. This type I error level was calculated using Bonferroni’s method, dividing the standard type I error level of 0.05 by the number of tests performed (0.05/26 = 0.002).

## 3. Results

### 3.1. Participant Characteristics

Characteristics for the total sample and for 8th and 11th graders separately are communicated in Table 1. For the total sample (*n* = 4730, Weighted *n* = 710,731), students were 14.7 ± 1.6 years of age, 49.0% female, and 51.4% identified as Hispanic. Students with overweight/obesity comprised 43.7% of the sample. The average percent of economic disadvantage by the school was 66.4 ± 19.9% and the average percent of students with limited English proficiency by school was 13.0 ± 12.9%. The average SPAN HEI score was 47.7 ± 5.9 (scale of 0 to 100). Most students reported never (41.4%) or almost never (19.6%) using the Nutrition Facts labels to make their food choices, 11.0% reported almost always or always using the Nutrition Facts labels to make food choices, and the remaining 27.9% reported they sometimes used Nutrition Facts labels to make food choices. The number of times students reported eating individual food items is reported in Table S1.

### 3.2. Associations between Nutrition Label Usage and Eating Indices

**Figure 1:**
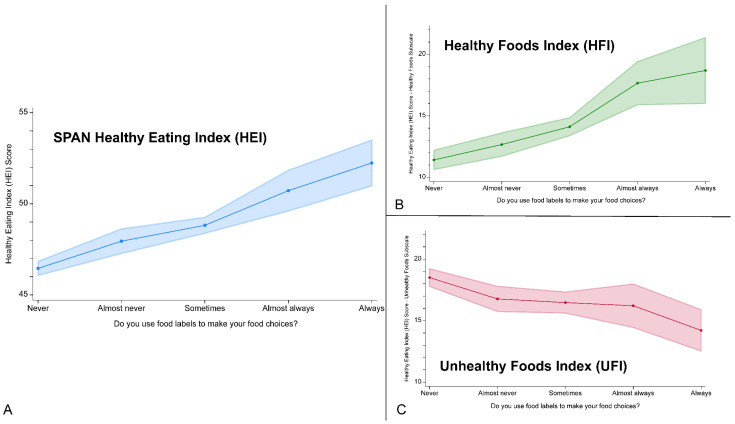
Marginal predicted means of (**A**) SPAN Healthy Eating Index (HEI), (**B**) Healthy Foods Index (HFI), and (**C**) Unhealthy Foods Index (UFI) associated with using food labels to make food choices among 8th and 11th-grade students in Texas. Note: Marginal predicted means calculated from weighted linear regression models communicated in Table 2. Note: The y-axis of the SPAN Healthy Eating Index (HEI) starts at 45 and the y-axis of the Healthy Foods Index (HFI) and the Unhealthy Foods Index (UFI) start at 10 for visualization.

Results from weighted (see Section 2.2) linear regression analyses predicting SPAN HEI, HFI, and UFI are summarized in Table 2. Compared to never using Nutrition Facts labels to make food choices, almost never, sometimes, almost always, and always using nutrition labels significantly and positively associated with SPAN HEI scores and HFI scores, with each increase in Nutrition Facts label usage corresponding to an increase in the strength of the association. Compared to never using nutrition labels to make food choices, almost never, sometimes, almost always, and always using nutrition labels was significantly and negatively associated with UFI scores, and a similar dose–response relationship was found. To illustrate this dose–response relationship more clearly, marginal predicted means of SPAN HEI, HFI, and UFI scores associated with using food labels to make food choices are visually displayed in Figure 1 and communicated numerically in Table S2.

### 3.3. Associations between Nutrition Label Usage and Individual Food Items

**Figure 2:**
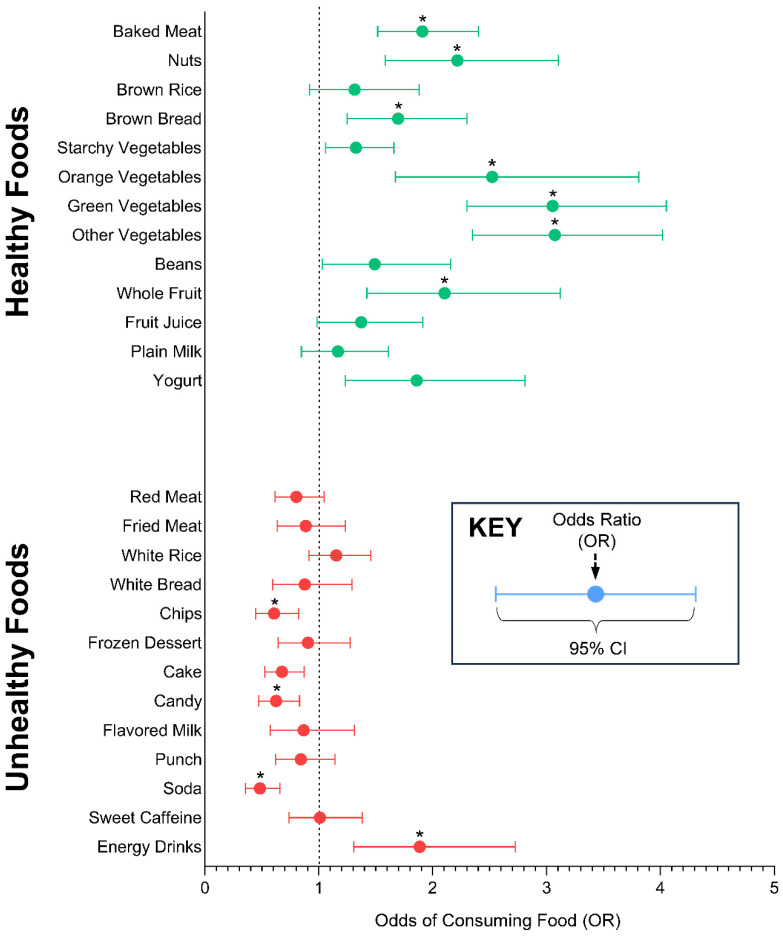
Odds of consuming healthy and unhealthy foods when always/almost always using food labels to make food choices. Note: Never/almost never using food labels served as the referent variable; Odds Ratios (ORs) calculated from separate weighted logistic regression models for each food item, adjusting for grade, sex, Body Mass Index (BMI), race/ethnicity, percent economic disadvantage by school, and percent limited English proficiency by school; * denotes significance at 0.002. Model estimates are provided in Table S3.

The odds of consuming 26 food items when using nutrition labels to make choices are visually communicated in Figure 2, separated into 13 healthy food items and 13 unhealthy food items. Full model estimates are provided in Table S3. Students who reported always/almost always using the Nutrition Facts labels to make food choices had significantly higher odds of consuming baked meat, nuts, brown bread, orange, green, and other vegetables, fruit, and energy drinks, and significantly lower odds of consuming chips, candy, and soda compared to those who reported never/almost using nutrition labels.

## 4. Discussion

We explored cross-sectional relationships between 8th and 11th-grade students’ use of the Nutrition Facts labels to make food choices and various aspects of their dietary behavior, including healthy and unhealthy eating. We found multiple indications that adolescents who used the Nutrition Facts labels to make food choices were more likely to consume several healthy food items and less likely to consume most unhealthy food items. Positive associations were seen between the frequency of nutrition label usage and HEI and HFI scores, i.e., adolescents who regularly engaged with the Nutrition Facts labels were more likely to make food choices aligned with healthier dietary patterns and greater intake of nutrient-dense foods. We also identified negative and significant dose–response relationships between nutrition label usage and UFI scores, which represent unhealthy food consumption, even after adjusting the analysis for socio-demographic factors. These findings were reflected in analyses of individual foods as well, including nutrient-dense foods such as baked meat, nuts, various vegetables, beans, yogurt, and fruit, as well as foods high in added sugars and unhealthy fats, including chips, cake, candy, and soda. Overall, our results are consistent with findings from studies in other populations [26], and with conclusions from systematic reviews [27].

While we did show that the Nutrition Facts label use is associated with healthier eating among adolescents, another primary finding in our study was the low frequency of nutrition label use among this age group. Only a few published studies from the past two decades report the prevalence of nutrition label usage in the US. For example, Ollberding et al. [4] found that more than half of US adults reported using nutrition labels on the 2005–2006 NHANES. Moreover, that study found that label users reported healthier nutrition consumption, which parallels findings from our study among adolescents. Another study found that less than a third of low-income adults report regularly reading nutrition labels at home or the grocery store [28], and other studies have identified some of the other personal and demographic characteristics that might be associated with the use of nutrition labels among adults, including sex and nutrition-related attitudes/beliefs [29]. Similar future studies should continue to explore these factors among younger populations. To our knowledge, very few studies have also reported the prevalence of nutrition label usage among adolescents in the US. Again, using data from the 2005–2006 NHANES, Wojcicki and Heyman [14] found that less than 25% of adolescents used nutrition labels to make their food choices. Specifically, Wojcicki and Heyman found that 62.4% of adolescents reported they did not or rarely used nutrition labels, which is very similar to our study (61.0% of adolescents reported never/almost never using nutrition labels). There are a few examples of this being carried out outside of the US with smaller, non-representative samples as well [30,31,32]. If our results are consistent with a causal effect of nutrition label usage on diet quality, the low prevalence of nutrition label usage underscores the potential for educational and public health interventions aimed at enhancing awareness and promoting the use of nutrition labels among this age group.

One of the concerns with the use of nutrition labels is the complexity of information that must be conveyed. Several authors have noted that nutrition labels do not live up to their potential to communicate nutrition information clearly [33]. Our demonstration that label usage is positively associated with healthy food consumption, and negatively associated with unhealthy food consumption, suggests that adolescents who read nutrition labels can apparently comprehend and respond to the entirety of the Nutrition Facts label, including information on nutrients that they should have less of (saturated fat, added sugars, sodium), as well as those that they should have more of (dietary fiber, nutrients, minerals) [34]. This could also be a result of the US FDA’s recent update to nutrition labels [1,2], which now displays serving sizes and calories in a larger and bolder font, requires added sugars to be included in grams and a percent Daily Value, updated the list of nutrients required/permitted on the label, and updated footnotes to better explain percent Daily Value information. Few studies have explored how these recent changes impact nutrition label usage, but findings from our study shed preliminary light on current nutrition label usage among adolescents. Future studies should continue to explore how adolescents interact with and comprehend nutrition labels to better inform nutrition literacy intervention efforts, as there have been promising intervention studies among adolescents that have shown nutritional educational interventions can be effective at increasing nutrition label comprehension [35,36,37,38].

A caveat that should be noted was our finding of the positive association between nutrition label usage and the consumption of energy drinks, which was the only unhealthy food item which positively associated with the use of nutrition labels. This finding suggests that label usage may not be infallible in regulating specific healthy dietary choices among adolescents. Habitually consumed foods, such as energy drinks, may be governed by much more compelling forces than label usage. For example, an experimental study found that price manipulation influences the purchase of energy drinks, such that increasing prices decreases purchasing, especially among youth [39]. In line with labels, the experimental study also found that the inclusion of warning labels on energy drinks deterred their purchase among youth shoppers. Indeed, the inclusion of warning labels on these types of food items has been highlighted as a potential mechanism to deter consumption [40,41]. It could also be that adolescents are looking specifically to consume caffeinated beverages, and since energy drinks are heavily marketed on social media, adolescents tend to choose them for consumption over other beverages [42,43,44,45,46]. Moving forward, it is worth identifying such foods in future research, so that we can understand the limits of nutrition labels as a mechanism of behavioral nutrition-related choices and explore ways to encourage adolescents to choose healthier options.

We also found significant and positive associations between nutrition label usage and both the healthy and unhealthy food index scores for boys when compared to girls. While we did not compare the percentage of boys versus girls who used labels to make food choices, other studies have found that significantly more girls than boys report reading/using nutrition labels [30] and that this trend tracks into adulthood [47], although the research on this is limited. Our results would suggest that boys and girls may use nutrition labels differently when making food choices and the subsequent impact of nutrition label usage on food choice differs between boys and girls. More research should be conducted on these sex-based differences to better inform intervention efforts that may be tailored to meet the specific needs of boys and girls.

Despite the few concerning findings of our study, including the low frequency of nutrition label use among adolescents and the positive association of nutrition label use with energy drink consumption, the overall outcome was that nutrition label use does indeed beneficially influence healthier dietary choices among this age group. The dose–response relationship between nutrition label use and healthier dietary choices further emphasizes the fact that even sometimes interacting with nutrition labels can be beneficial when compared to not using them at all. This is promising as it suggests even incremental progress within nutrition literacy and nutrition-related public health education efforts can positively contribute to healthier dietary choices among adolescents. Indeed, knowledge and motivation have been shown to be positively associated with using nutrition labels to make food choices [48]. Avenues for these nutrition-related public health education efforts could include those at the policy and school level [49,50], as well as family-based programs, as parental knowledge and eating behaviors have been shown to influence nutrition-related outcomes among youth [51,52,53,54,55]. Recent large-scale initiatives include front-of-pack labeling, which has been implemented in the U.S. [56] and several other countries including Mexico, Canada, Australia, Chile, and the United Kingdom [57]. These front-of-pack initiatives have shown promise to increase awareness, use, and understanding of nutrition labels among youth [57] and future efforts should be made to scale up these types of efforts to encourage label usage among children and adolescents.

## 5. Strengths and Limitations

Our study on nutrition label usage and eating behaviors of adolescents has several strengths including the statewide representativeness of the data, the large and diverse sample size, and the inclusion of 26 individual food items used to create composite healthy and unhealthy eating indices as well as single item analyses. To the authors’ knowledge, this is also one of the first studies to explore adolescents’ nutrition label usage after the changes to US food labels had been administered [1,2]. Limitations of this study are noted as well and should be considered when interpreting findings. First, these analyses are based on cross-sectional data and may reflect reverse causation or residual confounding. Nevertheless, the consistency of results across a variety of outcome measures and specifications of the label usage variable, as well as the plausibly low prevalence of label usage, provides some basis for inference of causality. Second, the ‘exposure’ variable of nutrition label usage is likely measured with a considerable amount of error. As mentioned earlier, reading, comprehending, and acting on the information on nutrition labels is a cognitively complex task that cannot be sufficiently captured with a single question, even one allowing for five response options. However, the effect of imprecise measurement of exposure would be to bias associations towards the null. Despite this potential for bias, our results are consistently positive across multiple dietary outcomes. Third, our use of self-reported dietary instruments is a weakness. Such instruments are prone to inaccuracies resulting from social desirability bias and recall bias [58]. Recall bias was attenuated by asking participants to report what they ate/drank the previous day, but overall, our data limits any causal or longitudinal interpretations. There may also be unintended confusion surrounding some of the questions. For example, the dietary measure categorizes baked meat as healthy, but red meat as unhealthy. So, if participants had eaten baked red meat, those participants may have had trouble answering this question. Additionally, the sample was limited to 8th and 11th-grade students in Texas, which reduces the generalizability of the findings both in age group and geographic location, although 10% of children in the U.S. live in Texas and the demographics of Texas are a bellwether for demographic trends in the U.S. Finally, some food items assessed in this study might not necessarily be packaged with nutrition labels (e.g., whole fruits and vegetables). Even though we did find significant and positive associations between nutrition label usage and consumption of likely unpackaged (and perhaps unlabeled) food items, future studies exploring mechanisms between the use of nutrition labels and certain food items are warranted.

## 6. Conclusions

Using Nutrition Facts labels to make food choices is beneficially associated with healthy and unhealthy eating among 8th and 11th-grade students in Texas. These findings align with the notion that nutrition labels serve as valuable and practical tools guiding adolescents toward making more balanced and nutritious food choices. It emphasizes the potential benefits of instilling nutrition label literacy among adolescents through targeted interventions and education. However, the proportion of students using nutrition labels to make their food choices was low in our sample of 8th and 11th-grade students in Texas, which underscores the need for nutrition literacy education initiatives among middle and high school students. Future studies should explore both the prevalence of nutrition label usage among adolescents (and younger populations, including children) at a broader level and how usage may be associated with dietary behaviors and other important health outcomes, including overweight and obesity. High-quality evidence supporting the potential links between nutrition label usage, dietary behaviors, and health outcomes, including overweight and obesity, may inform obesity-related behavioral interventions for adolescents. Finally, public health efforts should be made to improve nutrition literacy and encourage nutrition label use among students in the U.S.

## Figures and Tables

**Table 1:** Demographic characteristics and nutrition-related behavioral variables presented as unweighted count/mean and weighted prevalence for the total sample of 8th and 11th graders (2019–2020 SPAN).

Characteristics	Total *n* = 4730 Weighted *n* = 710,731		8th Grade *n* = 2789 Weighted *n* = 369,248		11th Grade *n* = 1941 Weighted *n* = 341,483	
	Unweighted Count/Mean (SD)	Weighted Percent (%)	Unweighted Count/ Mean (SD)	Weighted Percent (%)	Unweighted Count/ Mean (SD)	Weighted Percent (%)
Age (years)	14.7 (1.6)	-	13.4 (0.6)		16.4 (0.5)	
Sex (female)	2369	49.0	1371	48.6	998	49.5
Race/Ethnicity						
African American	644	12.5	401	12.6	240	12.4
Hispanic	2514	51.4	1494	51.9	1020	50.9
White/Other	1575	36.1	894	35.5	681	36.7
Overweight/Obesity Status						
Healthy Weight	2530	56.3	1494	54.0	1036	58.8
Overweight	912	19.2	579	21.2	33	17.0
Obese	1288	24.5	716	24.8	572	24.2
Percent economically disadvantaged by school (%)	66.4 (19.9)	-	67.9 (20.4)	-	64.3 (18.9)	-
Percent limited English proficiency by school (%)	13.0 (12.9)	-	14.4 (14.5)	-	11.5 (8.7)	-
SPAN Healthy Eating Index (HEI)	47.9 (5.9)	-	48.0 (6.1)	-	47.9 (5.6)	-
Food label usage to make food choices						
Never	1975	41.4	1151	41.6	824	41.3
Almost Never	899	19.6	546	21.1	353	17.9
Sometimes	1302	27.9	751	27.3	551	28.6
Almost always	265	6.0	148	5.1	117	7.1
Always	222	5.0	141	4.9	81	5.2

**Table 2:** Summary of weighted linear regression predicting SPAN Healthy Eating Index (HEI), Healthy Foods Index (HFI), and Unhealthy Foods Index (UFI) for the total sample of 8th and 11th-grade students (*n* = 4414, weighted *n* = 694,656).

Predictor	b-Coefficient	95%CI	*p*-Value
**SPAN Healthy Eating Index (HEI)**			
Food label usage to make food choices (“Never” referent)			
Almost Never	**1.49**	0.74, 2.25	<0.001
Sometimes	**2.37**	1.79, 2.95	<0.001
Almost Always	**4.27**	3.16, 5.38	<0.001
Always	**5.79**	4.45, 7.12	<0.001
Grade (8th-grade referent)	−0.12	−0.70, 0.45	0.68
Sex (Female referent)	0.34	−0.16, 0.84	0.18
BMI Classification (Healthy weight referent)			
Overweight	0.30	−0.25, 0.85	0.28
Obese	0.28	−0.41, 0.96	0.42
Race/Ethnicity (White/Other referent)			
African American	−0.63	−1.62, 0.36	0.21
Hispanic	0.63	−0.08, 1.36	0.08
Percent economically disadvantaged (Lower economic disadvantage referent)			
Middle economic disadvantage	**−0.86**	−1.67, −0.05	0.04
Higher economic disadvantage	**−1.68**	−2.69, −0.68	0.001
Percent limited English proficiency	**0.03**	0.01, 0.06	0.04
Intercept	**46.37**	45.50, 47.24	<0.001
**Healthy Foods Index (HFI)**			
Food label usage to make food choices (“Never” referent)			
Almost Never	**1.25**	0.04, 2.46	0.04
Sometimes	**2.70**	1.62, 3.78	<0.001
Almost Always	**6.24**	4.37, 8.12	<0.001
Always	**7.28**	4.48, 10.07	<0.001
Grade (8th-grade referent)	**−1.07**	−2.05, −0.09	0.03
Sex (Female referent)	**2.53**	1.53, 3.53	<0.001
BMI classification (Healthy weight referent)			
Overweight	−0.71	−1.68, 0.26	0.15
Obese	**−1.34**	−2.36, −0.32	0.01
Race/Ethnicity (White/Other referent)			
African American	−0.07	−1.80, 1.67	0.94
Hispanic	0.42	−0.81, 1.66	0.50
Percent economically disadvantaged (Lower economic disadvantage referent)			
Middle economic disadvantage	**−1.23**	−2.46, −0.01	0.05
Higher economic disadvantage	**−1.29**	−2.43, −0.14	0.03
Percent limited English proficiency	0.01	−0.04, 0.05	0.95
Intercept	**11.66**	10.38, 12.95	<0.001
**Unhealthy Foods Index (UFI)**			
Food label usage to make food choices (“Never” referent)			
Almost Never	**−1.74**	−2.85, −0.62	0.003
Sometimes	**−2.03**	−3.07, −0.99	<0.001
Almost Always	**−2.29**	−4.08, −0.50	0.01
Always	**−4.30**	−6.25, −2.34	<0.001
Grade (8th-grade referent)	−0.83	−1.91, 0.26	0.13
Sex (Female referent)	**1.85**	1.07, 2.62	<0.001
BMI classification (Healthy Weight referent)			
Overweight	**−1.31**	−2.33, −0.29	0.01
Obese	**−1.90**	−2.88, −0.91	<0.001
Race/Ethnicity (White/Other referent)			
African American	1.20	−0.66, 3.07	0.20
Hispanic	−0.84	−2.08, 0.40	0.18
Percent economically disadvantaged (Lower economic disadvantage referent)			
Middle economic disadvantage	0.49	−0.73, 1.70	0.43
Higher economic disadvantage	**2.08**	0.52, 3.64	0.01
Percent limited English proficiency	**−0.06**	−0.12, −0.01	0.03
Intercept	**18.93**	17.63, 20.22	<0.001

## Data Availability

The datasets used and analyzed during the current study are available from the corresponding author on reasonable request and also available via the Texas SPAN Data Explorer: https://span-interactive.sph.uth.edu/, accessed on 10 July 2023.

## Supplementary Materials

The following supporting information can be downloaded at: https://www.mdpi.com/article/10.3390/nu16020311/s1, Table S1: Adolescents’ self-reported prior day eating habits of 26 individual food items from the 2019–2020 Texas SPAN survey communicated as weighted percentages; Table S2: Marginal predicted probabilities of SPAN HEI, HFI, and UFI scores associated with nutrition label usage calculated post-hoc from linear regression models, adjusted for grade, sex, Body Mass Index (BMI), race/ethnicity, economic disadvantage, and percent limited English proficiency by school. Table S3: Logistic regression results for the odds of consuming individual food items during the prior day when using nutrition labels to make food choices. Note: All models run separately for each food item and adjusted for grade, sex, Body Mass Index (BMI), race/ethnicity, economic disadvantage, and percent limited English proficiency by school.

## List of Abbreviations

BMI	Body Mass Index
CDC	Centers for Disease Control and Prevention
FDA	Food and Drug Administration
HFI	Healthy Foods Index
HSR	Health Service Region
SPAN HEI	SPAN Healthy Eating Index
TEA	Texas Education Agency
Texas SPAN	Texas School Physical Activity and Nutrition survey
UFI	Unhealthy Foods Index
US	United States
USDA	United States Department of Agriculture
