# Supplementary material for: Using the Nutrition Facts Label to Make Food Choices Is Associated with Healthier Eating among 8th and 11th-Grade Students: An Analysis of Statewide Representative Data from the 2019–2020 Texas School Physical Activity and Nutrition Survey
Source: Nutrients. 2024 Jan 20;16(2):311. doi: 10.3390/nu16020311 (PMC10819117; doi:10.3390/nu16020311)
Supplement: Supplementary file 1 [file nutrients-16-00311-s001.zip › nutrients-2808308-supplementary.pdf]

nutrients MDPI

Table S1. Adolescents' self-reported prior day eating habits of 26 individual food items from the 2019–2020 Texas SPAN survey communicated as weighted percentages.

Food Item	Frequency			
	0 Times (%)	1 Time (%)	2 Times (%)	3 or More (%)
	**Healthy Foods**			
Baked Meat	71.5	24.0	3.5	1.1
Nuts	71.7	21.8	4.8	1.7
Brown Rice	83.6	14.1	2.0	0.3
Brown Bread	68.2	24.3	6.1	1.4
Starchy Vegetables	71.8	22.5	4.4	1.3
Orange Vegetables	74.3	20.4	4.1	2.1
Green Vegetables	71.1	23.4	4.0	1.5
Other Vegetables	65.1	26.3	6.5	2.1
Beans	80.4	16.2	2.4	1.1
Whole Fruit	39.5	38.9	15.2	6.4
Fruit Juice	64.0	27.0	6.0	3.0
Plain Milk	60.0	28.5	8.2	3.4
Yogurt	86.5	11.4	1.4	0.7
	**Unhealthy Foods**			
Red Meat	47.8	39.3	10.9	2.0
Fried Meat	63.2	30.0	5.5	1.3
White Rice	62.7	30.8	5.4	1.1
White Bread	39.5	41.2	15.1	4.2
Chips	24.1	49.0	12.5	4.3
Frozen Dessert	78.4	18.3	2.4	0.9
Cake	57.2	33.2	7.3	2.4
Candy	55.4	31.1	7.0	6.4
Flavored Milk	73.8	20.2	4.8	1.2
Punch	61.0	27.9	7.6	3.4
Soda	55.1	33.7	8.0	3.3
Sweet Caffeine	73.1	19.8	4.8	2.3
Energy Drinks	88.4	9.3	1.5	0.8

Table S2. Marginal predicted probabilities of SPAN HEI, HFI, and UFI scores associated with nutrition label usage calculated post-hoc from linear regression models, adjusted for grade, sex, Body Mass Index (BMI), race/ethnicity, economic disadvantage, and percent limited English proficiency by school.

Food label usage to make food choices	Margin	95% CI	p-value
SPAN Healthy Eating Index (HEI)			
Never	45.5	46.1, 46.8	<0.001
Almost never	47.9	47.3, 48.6	<0.001
Sometimes	48.8	48.4, 49.3	<0.001
Almost always	50.7	49.6, 51.8	<0.001
Always	52.2	51.0, 53.5	<0.001
Healthy Foods Index (HFI)			
Never	11.4	10.6, 12.2	<0.001
Almost never	12.7	11.7, 13.6	<0.001
Sometimes	14.1	13.4, 14.8	<0.001
Almost always	17.7	15.9, 19.4	<0.001
Always	18.7	16.0, 21.4	<0.001
Unhealthy Foods Index (UFI)			
Never	18.5	17.8, 19.2	<0.001
Almost never	16.8	15.7, 17.8	<0.001
Sometimes	16.5	15.6, 17.3	<0.001
Almost always	16.2	14.4, 18.0	<0.001

Always	14.2	12.5, 15.9	<0.001

Table S3. Logistic regression results for the odds of consuming individual food items during the prior day when using nutrition labels to make food choices. Note: All models run separately for each food item and adjusted for grade, sex, Body Mass Index (BMI), race/ethnicity, economic disadvantage, and percent limited English proficiency by school.

Food Label Usage to Make Food Choices ("Never/Almost Never" Referent)	Odds Ratio (OR)	95%CI	*p*-Value
**Baked Meat**			
Sometimes	1.30	1.08,1.55	0.005
Always/Almost always	1.91	1.52,2.40	<0.001
**Nuts**			
Sometimes	1.27	1.01,1.58	0.04
Always/Almost always	2.22	1.58,3.10	<0.001
**Brown Rice**			
Sometimes	0.98	0.74,1.27	0.85
Always/Almost always	1.31	0.92,1.88	0.13
**Brown Bread**			
Sometimes	1.24	1.01,1.51	0.04
Always/Almost always	1.70	1.25,2.30	0.001
**Starchy Vegetables**			
Sometimes	1.25	0.99,1.59	0.06
Always/Almost always	1.33	1.06,1.66	0.01
**Orange Vegetables**			
Sometimes	1.92	1.47,2.51	<0.001
Always/Almost always	2.52	1.67,3.81	<0.001
**Green Vegetables**			
Sometimes	1.66	1.30,2.10	<0.001
Always/Almost always	3.05	2.30,4.05	<0.001
**Other Vegetables**			
Sometimes	1.62	1.31,1.99	<0.001
Always/Almost always	3.07	2.35,4.02	<0.001
**Beans**			
Sometimes	1.26	0.99,1.61	0.07
Always/Almost always	1.49	1.03,2.16	0.03
**Whole Fruit**			
Sometimes	1.65	1.31,2.08	<0.001
Always/Almost always	2.11	1.42,3.12	<0.001
**Fruit Juice**			
Sometimes	1.10	0.86,1.42	0.45
Always/Almost always	1.37	0.99,1.91	0.06
**Plain Milk**			
Sometimes	1.30	1.11,1.52	0.001
Always/Almost always	1.17	0.85,1.61	0.34
**Yogurt**			
Sometimes	1.14	0.87,1.49	0.34
Always/Almost always	1.86	1.23,2.81	0.004
**Red Meat**			
Sometimes	1.06	0.87,1.29	0.55
Always/Almost always	0.80	0.61,1.04	0.11
**Fried Meat**			
Sometimes	0.96	0.79,1.18	0.72
Always/Almost always	0.88	0.63,1.23	0.47
**White Rice**			
Sometimes	1.06	0.86,1.30	0.58
Always/Almost always	1.15	0.91,1.46	0.23

Food Label Usage to Make Food Choices (“Never/Almost Never” Referent)	Odds Ratio (OR)	95%CI	*p*-Value
**White Bread**			
Sometimes	1.09	0.85,1.41	0.48
Always/Almost always	0.88	0.60,1.29	0.50
**Chips**			
Sometimes	0.90	0.76,1.06	0.20
Always/Almost always	0.61	0.45,0.82	0.002
**Frozen Dessert**			
Sometimes	0.09	0.77,1.14	0.52
Always/Almost always	0.91	0.64,1.27	0.57
**Cake**			
Sometimes	0.87	0.68,1.10	0.24
Always/Almost always	0.68	0.52,0.87	0.003
**Candy**			
Sometimes	0.77	0.60,0.99	0.04
Always/Almost always	0.63	0.47,0.83	0.001
**Flavored Milk**			
Sometimes	1.04	0.82,1.32	0.73
Always/Almost always	0.87	0.57,1.31	0.50
**Punch**			
Sometimes	0.96	0.77,1.21	0.74
Always/Almost always	0.84	0.62,1.14	0.26
**Soda**			
Sometimes	0.68	0.58,0.80	<0.001
Always/Almost always	0.48	0.36,0.66	<0.001
**Sweet Caffeine**			
Sometimes	0.87	0.65,1.16	0.34
Always/Almost always	1.01	0.74,1.38	0.95
**Energy Drinks**			
Sometimes	0.98	0.72,1.33	0.89
Always/Almost always	1.89	1.31,2.72	0.001

Disclaimer/Publisher’s Note: The statements, opinions and data contained in all publications are solely those of the individual author(s) and contributor(s) and not of MDPI and/or the editor(s). MDPI and/or the editor(s) disclaim responsibility for any injury to people or property resulting from any ideas, methods, instructions or products referred to in the content.
